# Lack of Fractalkine Receptor on Macrophages Impairs Spontaneous Recovery of Ribbon Synapses After Moderate Noise Trauma in C57BL/6 Mice

**DOI:** 10.3389/fnins.2019.00620

**Published:** 2019-06-13

**Authors:** Tejbeer Kaur, Anna C. Clayman, Andrew J. Nash, Angela D. Schrader, Mark E. Warchol, Kevin K. Ohlemiller

**Affiliations:** ^1^Department of Otolaryngology, Washington University School of Medicine, St. Louis, MO, United States; ^2^Program in Audiology and Communication Sciences, Washington University School of Medicine, St. Louis, MO, United States

**Keywords:** cochlea, ribbon synapses, noise-induced hearing loss, macrophages, fractalkine, C57BL/6 mice

## Abstract

Noise trauma causes loss of synaptic connections between cochlear inner hair cells (IHCs) and the spiral ganglion neurons (SGNs). Such synaptic loss can trigger slow and progressive degeneration of SGNs. Macrophage fractalkine signaling is critical for neuron survival in the injured cochlea, but its role in cochlear synaptopathy is unknown. Fractalkine, a chemokine, is constitutively expressed by SGNs and signals via its receptor CX_3_CR1 that is expressed on macrophages. The present study characterized the immune response and examined the function of fractalkine signaling in degeneration and repair of cochlear synapses following noise trauma. Adult mice wild type, heterozygous and knockout for CX_3_CR1 on a C57BL/6 background were exposed for 2 h to an octave band noise at 90 dB SPL. Noise exposure caused temporary shifts in hearing thresholds without any evident loss of hair cells in CX_3_CR1 heterozygous mice that have intact fractalkine signaling. Enhanced macrophage migration toward the IHC-synaptic region was observed immediately after exposure in all genotypes. Synaptic immunolabeling revealed a rapid loss of ribbon synapses throughout the basal turn of the cochlea of all genotypes. The damaged synapses spontaneously recovered in mice with intact CX_3_CR1. However, CX_3_CR1 knockout (KO) animals displayed enhanced synaptic degeneration that correlated with attenuated suprathreshold neural responses at higher frequencies. Exposed CX_3_CR1 KO mice also exhibited increased loss of IHCs and SGN cell bodies compared to exposed heterozygous mice. These results indicate that macrophages can promote repair of damaged synapses after moderate noise trauma and that repair requires fractalkine signaling.

## Introduction

The inner hair cell-spiral ganglion neuron (IHC-SGN) synaptic transmission is excitatory and glutamatergic (Puel, 1995). Glutamate is released from IHCs (Ruel et al., 2008; Seal et al., 2008) and both NMDA- and AMPA-type glutamate receptors are present on SGNs (Eybalin, 1993). The IHC-SGN synapses (a.k.a ribbon synapses) are vulnerable to degeneration due to noise trauma (Puel et al., 1998). Such degeneration has been attributed to glutamate excitotoxicity. Excessive glutamate release due to acoustic trauma can overstimulate postsynaptic glutamate receptors on afferent nerve fiber (ANF) terminals resulting in their swelling, disruption of postsynaptic structures, degeneration of terminals and loss of function. Such synaptic damage can be blocked by glutamate receptor antagonists (Pujol et al., 1985, 1993; Gil-Loyzaga and Pujol, 1990; Puel et al., 1994, 1998; Ruel et al., 2007), while inhibiting glutamate reuptake exacerbates damage (Hakuba et al., 2000). Synaptic degeneration can precede both hair cell loss and threshold elevation and can trigger gradual loss of SGNs (Kujawa and Liberman, 2009). Such primary neural degeneration can affect sound localization and understanding of speech in noisy environments (Liberman, 2017). Moreover, lack of SGNs can limit the effectiveness of primary therapies for hearing loss such as hearing aids and cochlear implants. The denervated IHCs can be partially reinnervated by SGNs after excitotoxic or acoustic trauma; (Pujol et al., 1985; Puel et al., 1995, 1998; Pujol and Puel, 1999; Wang and Green, 2011) however, the underlying mechanisms remain unclear.

Excitotoxicity has been involved in many central nervous system (CNS) acute and chronic neurodegenerative diseases including epilepsy, Alzheimer’s, Parkinson’s, stroke, and multiple sclerosis. CNS excitotoxicity can activate and recruit microglia (brain macrophages) to the site of injury and these microglia can protect neurons and improve synaptic recovery following excitotoxic damage (Simard and Rivest, 2007; Lauro et al., 2010; Vinet et al., 2012; Eyo et al., 2014; Kato et al., 2016). Microglia-mediated protection against excitotoxicity has been attributed to fractalkine signaling (Meucci et al., 1998; Chapman et al., 2000; Deiva et al., 2004; Limatola et al., 2005; Ragozzino et al., 2006; Lauro et al., 2008, 2015; Cipriani et al., 2011; Catalano et al., 2013; Roseti et al., 2013). Fractalkine signaling represents a unique immune-neuron receptor-ligand pair, where fractalkine (CX_3_CL1), a chemokine, is constitutively expressed on neurons in the CNS (Harrison et al., 1998; Kim et al., 2011) and by SGNs of mouse (Kaur et al., 2015) and human (Liu et al., 2018) cochlea. Fractalkine binds to its exclusive G-protein coupled receptor, CX_3_CR1, which is expressed by cochlear macrophages, microglia and peripheral leukocytes (Jung et al., 2000; Hirose et al., 2005). Fractalkine occurs in two different forms: as a membrane-bound protein tethered to neuronal membranes by a mucin-like stalk, and as a soluble factor released upon cleavage of its N-terminal chemokine domain by metalloproteases (ADAM10/ADAM17) (Imai et al., 1997; Garton et al., 2001). The soluble chemokine domain of fractalkine, when cleaved, can act as chemoattractant, while the membrane-tethered mucin-stalk of fractalkine has been proposed to act as an adhesion molecule for leukocytes during inflammation (Haskell et al., 1999; Hermand et al., 2008).

We previously demonstrated that macrophages promote the survival of SGNs via fractalkine signaling after loss of their target hair cells (Kaur et al., 2015, 2018). Notably, the role of macrophages and fractalkine signaling in degeneration and repair of synapses are unknown. The present study characterized the immune response and examined the contribution of fractalkine signaling toward degeneration and repair of damaged synapses after synaptopathic noise trauma. We report that moderate noise trauma caused rapid degeneration of ribbon synapses and immediate macrophage migration into the damaged synaptic region without any evident hair cell loss. The damaged synapses undergo post-exposure spontaneous recovery in animals with intact fractalkine signaling. Notably, disruption of fractalkine signaling diminished synaptic recovery and increased neuronal loss after noise trauma. To our knowledge this is the first evidence of a protective role for macrophages and fractalkine signaling in noise-induced cochlear synaptopathy.

## Materials and Methods

### Animals

The study used young adult (6 weeks of age) mice of both sexes on a C57BL/6 (B6) background. To define the role of fractalkine signaling in cochlear excitotoxicity CX_3_CR1^+/+^, CX_3_CR1^GFP/+^, and CX_3_CR1^GFP/GFP^ mice were employed (Jung et al., 2000). The mice were obtained from Dr. Hirose, Washington University in St. Louis, Missouri, originally obtained from Dan Littmann, New York University, New York. A targeted deletion of CX_3_CR1 and replacement with the gene encoding green fluorescent protein (GFP) rendered all monocytes and macrophages endogenously fluorescent (Jung et al., 2000), which facilitates their visualization through confocal microscope. Cochlear macrophages express CX_3_CR1 (Hirose et al., 2005). The CX_3_CR1^GFP/+^ mice (denoted as CX_3_CR1^+/-^ in the manuscript) with one copy of CX_3_CR1 retain fractalkine signaling, while CX_3_CR1^GFP/GFP^ mice (denoted as CX_3_CR1^-/-^ in the manuscript) lack fractalkine signaling. Identification of CX_3_CR1^+/-^ and CX_3_CR1^-/-^ followed previously described methods (Jung et al., 2000). Mice were housed in the animal facility at the Central Institute for the Deaf (Washington University School of Medicine) and were maintained on a 12 h/day-night light cycle with open access to food and water. All experimental protocols were approved by the Animal Studies Committee of the Washington University School of Medicine (St. Louis, MO, United States).

### Noise Exposures

The study employed mice on B6 background, which is a strain susceptible to noise- and age-related hearing loss (Henry and Chole, 1980; Hequembourg and Liberman, 2001). To induce cochlear synaptopathy, young mice (6 weeks of age) were exposed for 2 h to an octave 8–16 kHz band noise at 90 dB SPL. Noise exposures were performed in a foam-lined, single-walled soundproof room from Industrial Acoustics Company (IAC). Fully awake and unrestrained animals were placed singly or in pairs in modified cages (food, water, bedding removed) positioned up to two cages at once directly under an exponential horn. All noise was octave band (8–16 kHz), generated digitally using custom Labview routines (running on a PC) in conjunction with a Tucker-Davis Technologies Rz6 signal processor, and a Crown D-150A power amplifier that drove the speaker.

### Auditory Brainstem Response

All auditory brainstem responses (ABRs) were performed by a “blinded” observer. ABRs were analyzed both prior to noise exposure (pre-NE, baseline) and after noise exposure (post-NE) at time 0 h (immediately after noise exposure), 2 and 8 weeks (2 months) recovery. Mice were anesthetized via i.p. injections of ketamine (100 mg/kg) and xylazine (20 mg/kg). Subcutaneous electrodes were placed behind the right pinna (inverting) and vertex (active). A ground electrode was placed near the trail of the mouse. Stimuli were 5-ms tone pips (0.5 ms cos2 rise-fall), delivered at 21/s with alternating stimulus polarity. Recorded electrical responses were amplified (∼10,000X), filtered (300 Hz to 3 kHz) and averaged using BioSig software (Tucker-Davis Technologies, Alachua, FL). The sound level was decreased in 5-dB steps from 99 dB SPL down to 15 dB SPL. At each sound level, 1,024 responses were averaged, and response waveforms were discarded as artifacts if the peak-to-peak voltage exceeded 15 μV. Thresholds at 5, 10, 20, 28.3, 40, and 56.6 kHz were determined by a single observer who noted the lowest sound level at which a recognizable waveform could be obtained. Waveforms were confirmed as auditory-evoked responses by their increasing latency and decreasing amplitude as the intensity of the stimulus was lowered. These threshold values (actual or assigned) were then used to calculate the mean ABR thresholds at each stimulus frequency.

### Input/Output Function

For neural response, ABR wave 1 component was identified and the peak to trough amplitudes were computed by off-line analysis of stored ABR waveforms. ABR Wave I amplitude-versus-stimulus level (ABR I/O) data were obtained at 10 and 28.3 kHz. To minimize fatigue, repetition numbers varied from 100 at high sound levels to 1,000 near threshold. Stimuli were ordered from high to low sound levels [from max sound pressure available (∼100 dB) to 5 dB below visual detection of Wave I] in 5 dB steps. Wave I amplitude was measured from the estimated baseline prior to the response to the positive peak of Wave I. ABR wave 1 amplitudes were analyzed both prior to noise exposure (pre-NE, baseline), and after noise exposure (post-NE) at time 0 h (immediately after noise exposure), 2 and 8 weeks (2 months) recovery.

### Distortion Product Otoacoustic Emissions

Mice were anesthetized as described above. Stimuli were presented at 5–40 kHz and delivered to the right ear by a custom-coupling insert. Distortion product (DP) grams were obtained for f2 ranging from 5 to 40 kHz, with a frequency ratio of f2/f1 of 1.2 and L1–L2 = 10 dB. Recordings were performed using EMAV software (Boys Town National Research Hospital). DPOAEs were performed by genotype-blinded person prior to noise exposure (pre-NE, baseline) and after noise exposure (post-NE) at time 0 h (immediately after noise exposure), 2 and 8 weeks (2 months) recovery.

### Histology

Mice were sacrificed at time 0 h, 24 h (1 day), 2 and 8 weeks after noise exposure for histology unless specified. Mice were deeply anesthetized with Fatal Plus (Sodium Pentobarbital) and perfused (intra-cardiac) with phosphate-buffered 4% paraformaldehyde (PFA) (Electron Microscopy Sciences). Temporal bones were removed and post-fixed in 4% PFA for 15 min on ice (for synaptic immunolabeling) or 1 h at room temperature (for hair cells, macrophages and neuron labeling), rinsed in phosphate-buffered saline (PBS) twice, and placed in 0.1 M Ethylenediaminetetraacetic acid (EDTA), to allow decalcification for whole-mount dissections and for frozen mid-modiolar sectioning. Proteins were detected in both cochlear surface preparations and in cochlear mid-modiolar frozen sections using standard immunofluorescence methods. Briefly, tissue was rinsed with PBS (three times) and incubated at room temperature for 2 h in blocking solution (5% normal horse serum in 0.2% Triton X-100 in PBS). Cochleae were incubated overnight at room temperature with combinations of the primary antibodies. Hair cells were labeled with antibody against Myosin VIIa (Proteus Biosciences, Cat. No. 25-6790, 1:500). Neurons were labeled using Neurofilament 165 (NF165, Developmental Studies Hybridoma Bank, Cat. No. 2H3C, 2 μg/ml) and Beta III Tubulin (β-III tubulin, Tuj-1) antibodies (Covance, Cat. No. MMS435P, 1:500). Macrophages are endogenously GFP positive in both CX_3_CR1^GFP/+^ and CX_3_CR1^GFP/GFP^ mice, however, to enhance the fluorescence signal macrophages were immunolabeled with antibody against GFP (Invitrogen, Cat. No. A-11122,1:500). IHC ribbon synapses at the presynaptic zones were labeled with antibody against CtBP2 (BD Biosciences, Cat. No. 612044, 1:200) and postsynaptic densities with AMPA receptor GluA3 antibody (Santa Cruz Biotechnology, Cat. No. SC-7612, 1:50).

### Cellular Imaging and Analyses

Fluorescence imaging was performed using an LSM 700 confocal microscope (Zeiss). For all cochleae, Z-series images were obtained at 10× (4.5-micron z-step-seize), 20× (1-micron z-step-size), or 63× (0.3-micron z-step-size) objectives. Image processing and quantitative analysis were performed using Volocity 3D image analysis software (version 6.1.1, PerkinElmer) and ImageJ (Fiji) 1.47b (National Institutes of Health).

### Hair Cell Counts

Both inner and outer hair cells were identified by their immunoreactivity for Myosin VIIa. Hair cells were counted from the apical (5, 8, and 11 kHz), middle (16, 22, 28 kHz), basal (32 and 45 kHz) and hook (56 and 64 kHz) region of the cochlea unless otherwise stated. Data are expressed as percentage hair cell survival along the cochlear length.

### Macrophage Counts

To assess macrophages per 100 μm of sensory epithelium, GFP-labeled macrophages were counted in the organ of Corti from maximum intensity projections taken from the apical (5, 8, and 11 kHz), middle (16, 22, 28 kHz) and basal (32 and 45 kHz) region of cochlear whole mounts. Macrophages were also counted in the IHC basal region above the habenula using yz optical sectioning and 3D slice features on Volocity 3D image analysis software (version 6.1.1, PerkinElmer) and reported as macrophages in the IHC-basal region per 100 μm of sensory epithelium. Macrophages in spiral ganglia were counted from at least 5–6 mid-modiolar sections per cochlea and normalized to the cross-sectional area of the Rosenthal’s canal of the respective cochlear turn and averaged as number per 1,000 μm^2^.

### Spiral Ganglion Neuron Counts

Spiral ganglion counts were analyzed at 8 weeks (2 months), 16 weeks (4 months), and 24 weeks (6 months) after noise exposure. To assess the numbers of spiral ganglion cell bodies, NF165 and Tuj-1 labeled somata within Rosenthal’s canal were counted from the maximum intensity projections of each section. Cell bodies counted from 5 to 6 sections per cochlea were normalized to the cross-sectional area of Rosenthal’s canal per cochlear turn and averaged and reported as SGN density (per 1,000 μm^2^).

### Synaptic Counts

Confocal z-stacks were obtained using a high-resolution oil-immersion objective (63×) from 5, 8, 11, 16, 22, 28, 32, and 45 kHz regions in each case. Each stack spanned the entire synaptic pole of the hair cells in the z-dimensions, with z-step-size of 0.3 μm, from apical portion of the IHC to nerve terminal in the habenula perforata region. Maximum intensity projection images were exported to ImageJ (Fiji) 1.47b software and converted to black and white images. Juxtaposed pre- and post-synaptic punctae were counted manually from row of 10–12 IHCs along the lengths of the organ of Corti in the apical (5, 8, and 11 kHz), mid-apical and mid-basal (16, 22, 28 kHz), and basal (32 and 45 kHz) regions of each cochlea. Synaptic counts were divided by total number of surviving IHCs in the image and reported as synaptic ribbons per IHC.

### Statistical Analyses

All the data analyses and statistics were performed using Prism version 7.0a (GraphPad). Data are presented as mean ± SD. *t*-test, one way or two-way ANOVA was applied as appropriate. Significance main effects or interactions were followed by appropriate *post hoc* tests. Details on error bars, statistical analysis, degree of freedom, number of animals, experimental replicates can be found in results and figure legends section of the manuscript. Results were considered statistically significant when probability (*p*-values) of the appropriate statistical test was less than or equal to the significance level, alpha (α) = 0.05. *F* values for ANOVAs are reported as *F*_(degree of freedom numerator, degree of freedom denominator)_.

## Results

### Moderate Noise Trauma Induces Temporary Hearing Loss Without Any Hair Cell Death

**FIGURE 1:**
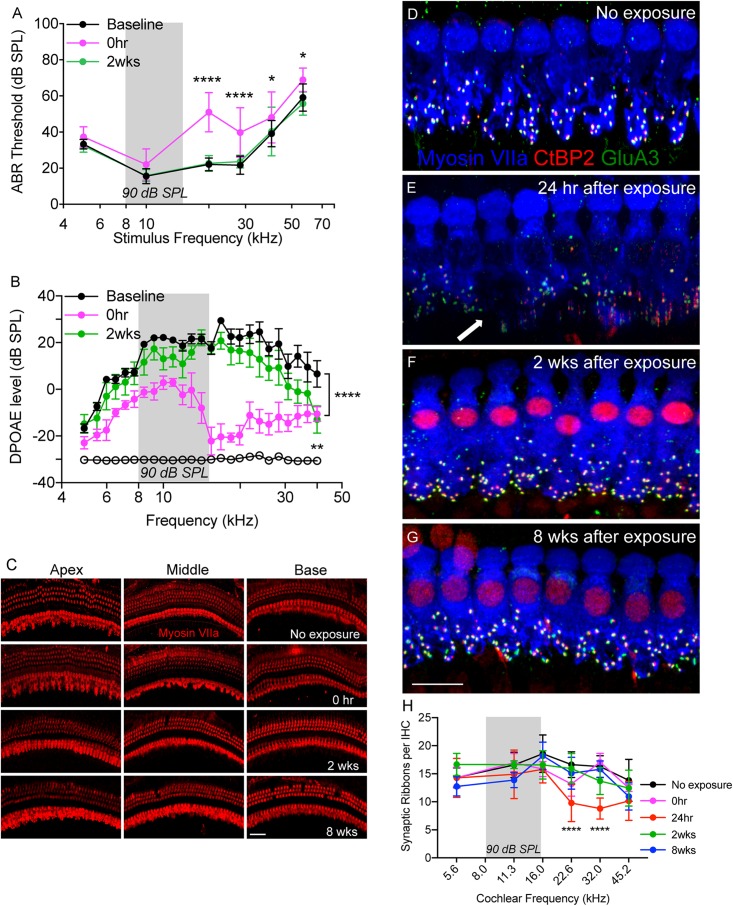
Temporary noise-induced hearing loss causes synaptopathy. **(A)** Auditory brainstem response thresholds plotted as a function of stimulus frequencies obtained from CX_3_CR1^+/-^ mice prior to noise exposure (baseline), and at 0 h and 2 weeks after noise exposure. *n* = 9, ^∗^*p* < 0.05 baseline vs. 0 h, ^∗∗∗∗^*p* < 0.0001 baseline vs. 0 h and 2 weeks. **(B)** DPgrams obtained from CX_3_CR1^+/-^ mice prior to noise exposure (baseline), and at 0 h and 2 weeks after noise exposure. *n* = 6, ^∗∗∗∗^*p* < 0.0001 baseline vs. 0 h, ^∗∗^*p* = 0.0046 baseline vs. 2 weeks (at 40 kHz). Open black circles represents noise floor. **(C)** Representative micrographs of cochlear whole mounts from apical, middle and basal regions of CX_3_CR1^+/-^ mice not exposed to noise (no exposure) and at time 0 h, 2 and 8 weeks recovery after exposure immunolabeled for hair cell marker, Myosin VIIa (red). **(D)** Representative micrograph from unexposed CX_3_CR1^+/-^ mouse cochlea at 32 kHz region immunolabeled for pre-synaptic marker CtBP2 (red), post-synaptic marker GluA3 (green) and inner hair cell marker Myosin VIIa (blue) showing intact juxtaposed ribbon synapses. **(E)** Representative micrograph from CX_3_CR1^+/-^ mouse cochlea at 24 h after exposure from 32 kHz region showing disintegrated ribbon synapses indicated by white arrow. **(F)** Representative micrograph from CX_3_CR1^+/-^ mouse cochlea at 2 weeks after exposure from 32 kHz region showing synaptic repair. **(G)** Representative micrograph from CX_3_CR1^+/-^ mouse cochlea at 8 weeks after exposure from 32 kHz region showing stable synapses. Labeling colors in **(E–G)** are same as reported in **(D)**. **(H)** Ribbon synapses per IHC along the cochlear length, *n* = 6–9 mice per time point, ^∗∗∗∗^*p* < 0.0001, 24 h vs. control. Gray bar in the graph represents the frequency band (8–16 kHz) of noise exposure. Scale bar, 63 μm **(C)** and 17 μm **(D–G)**.

CX_3_CR1^+/-^ mice (intact fractalkine signaling) were exposed for 2 h to moderate noise levels at 90 dB SPL. To verify whether such moderate noise exposure caused temporary or permanent hearing loss ABRs and DPOAEs were performed both prior to noise exposure (baseline) and after noise exposure (post-NE) at time 0 h (immediately after noise exposure), 2 and 8 weeks recovery. Noise exposure produced a ∼20–30 dB elevation of neural response thresholds at stimulus frequencies of 20, 28.3, 40, and 56.6 kHz at time 0 h recovery (Figure 1A). By 2 weeks post-exposure, response thresholds recovered to baseline thresholds and remained stable 8 weeks later (data not shown here). ABR thresholds at time 0 h recovery were significantly different at 20 and 28.3 kHz (*p* < 0.0001), and at 40 and 56.6 kHz (*p* = 0.01, *F*_(10,172)_ = 4.89, two-way ANOVA) when compared to thresholds at baseline and 2 weeks recovery. Elevation in ABR thresholds was coupled with attenuation of DPOAE levels at time 0 h recovery (Figure 1B) that nearly recovered to baseline DPOAE levels by 2 weeks recovery. DPOAE levels at time 0 h recovery were significantly different from baseline levels at frequencies above 6 kHz (*p* < 0.0001, *F*_(48,350)_ = 2.60, two-way ANOVA). DPOAE levels at 2 weeks were significantly different from baseline levels only at highest tested frequency (40 kHz) (*p* = 0.0046, two-way ANOVA). Moderate noise trauma did not result in loss of outer and IHCs throughout the cochlea at 0 h and 2 weeks after exposure, however, minimal degree of OHC loss was observed in the basal turn of the cochlea at 8 weeks after exposure (Figure 1C).

### Moderate Noise Trauma Induces Rapid Degeneration and Spontaneous Recovery of Synapses

We next examined whether such moderate noise trauma induces cochlear synaptopathy in CX_3_CR1^+/-^ mice. Synaptic immunolabeling and quantification revealed a rapid loss of synaptic ribbons in CX_3_CR1^+/-^ mice exposed to noise at time 24 h recovery throughout the mid-basal and basal cochlear region when compared to unexposed CX_3_CR1^+/-^ mice (Figure 1D,E,H). Ribbon synapses per IHC of unexposed CX_3_CR1^+/-^ mice were 14.32 ± 1.7 (5.6 kHz), 16.57 ± 2.2 (11.3 kHz), 18.57 ± 3.3 (16.0 kHz), 16.62 ± 2.2 (22.6 kHz), 16.28 ± 1.9 (32.0 kHz), and 13.82 ± 3.7 (45.2 kHz). Synaptic loss was evident as early as 0 h (Figure 1H) but was more robust at 24 h post-exposure. Ribbon synapses per IHC of CX_3_CR1^+/-^ mice at 24 h post-exposure were 14.26 ± 3.4 (5.6 kHz), 14.9 ± 4.3 (11.3 kHz), 15.8 ± 2.4 (16.0 kHz), 9.78 ± 3.3 (22.6 kHz), 8.79 ± 1.8 (32.0 kHz), and 10.16 ± 3.4 (45.2 kHz). Two-way ANOVA followed by Tukey’s multiple comparison tests revealed that synapses per IHC at 24 h post-exposure were statistically different from unexposed controls at 22.6 and 32.0 kHz (*p* < 0.0001, *F*_(20,149)_ = 2.62). The damaged synapses in exposed CX_3_CR1^+/-^ mice recovered by 2 weeks post-exposure and remained stable 8 weeks later (Figure 1F–H). Synapses per IHC of CX_3_CR1^+/-^ mice at time 2- and 8-weeks recovery were not different from that of unexposed CX_3_CR1^+/-^ mice (*p* > 0.05, Two-way ANOVA). Together, these results demonstrate that noise trauma at 90 dB SPL induces a temporary threshold shift (TTS) without any evident hair cell death and causes rapid synaptic degeneration that eventually repairs in young CX_3_CR1^+/-^ mice on B6 background.

### Noise Trauma Induces Immediate Macrophage Recruitment Into the IHC-Synaptic Region Despite No Hair Cell Loss

**FIGURE 2:**
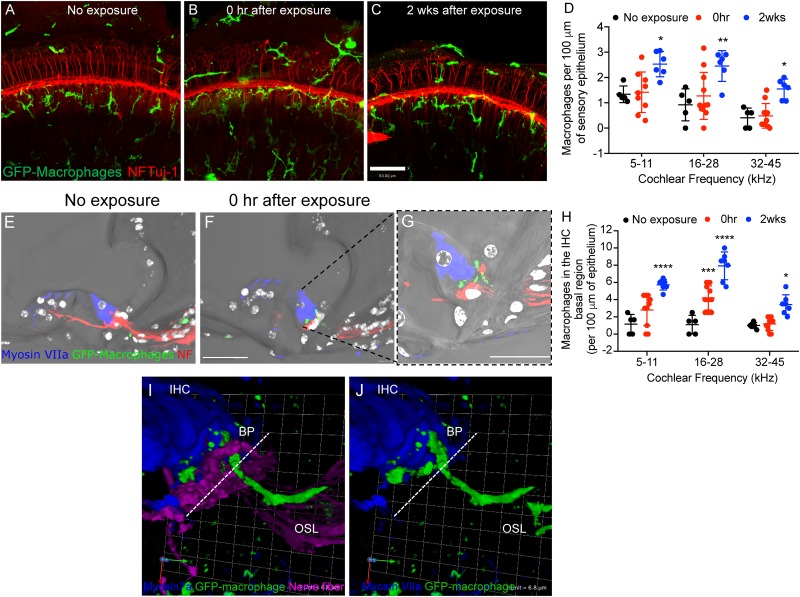
Macrophage migrate into the epithelium after noise trauma. Representative cochlear whole mount micrographs from CX_3_CR1^+/-^ mice **(A)** not exposed to noise (no exposure), **(B)** at 0 h, and **(C)** 2 weeks after noise exposure immunolabeled for macrophages (green, GFP) and peripheral nerve fibers (red, NF165 and Tuj-1). **(D)** Macrophage numbers per 100 μm of sensory epithelium. No exposure (*n* = 5), 0 h (*n* = 11) and 2 weeks (*n* = 7). ^∗^*p* < 0.05, ^∗∗^*p* < 0.01, control vs. 2 weeks. **(E)** The organ of Corti from an unexposed mouse (no exposure) showing macrophages in the osseous spiral lamina away from the IHC-synaptic region. **(F)** GFP-macrophage observed in the basal region of an inner hair cell (Myosin VIIa, blue) at 0 h after noise exposure. **(G)** Higher magnification of **(F)** showing GFP-macrophage above the habenula and in the basal region of the IHC at 0 h after synaptopathic noise exposure. White color in E-G represents nuclei stained with DAPI. **(H)** Macrophage numbers in the IHC basal region per 100 μm of epithelium. No exposure (*n* = 5), 0 h (*n* = 11) and 2 weeks (*n* = 7).^∗∗∗^*p* < 0.001 (16–28 kHz), no exposure vs. 0 h, ^∗∗∗∗^*p* < 0.0001 (5–11, 16–28 kHz), ^∗^*p* < 0.05 (32–45 kHz), no exposure vs. 2 weeks. **(I,J)** 3D reconstruction of a confocal stack showing pseudopodia (cytoplasmic extension) of a GFP-macrophage above the habenula and under the basal pole (BP) of an inner hair cell (IHC, blue) and the cell body of the macrophage in the osseous spiral lamina (OSL). Dashed line represents demarcation of the organ of Corti from the OSL. Scale bar, 63 μm **(A–C)**, 20 μm **(E,F)**, 10 μm **(G)**, and 6.8 μm **(I,J)**.

To determine whether moderate noise exposure that does not cause hair cell death is able to activate and increase macrophage numbers in the damaged epithelium, we examined GFP positive macrophages in the sensory epithelium as well as in the inner spiral plexus of CX_3_CR1^+/-^ mice not exposed to noise (no exposure) and at time 0 h and 2 weeks after TTS-like noise trauma. In unexposed CX_3_CR1^+/-^ mice, GFP-macrophages are occasionally observed below or in the basilar membrane and also in the osseous spiral lamina away from the inner spiral plexus (Figure 2A,D,E,H). Moderate noise trauma led to an immediate (0 h) increase in macrophage numbers in the apical and middle region of the epithelium (Figure 2B) and also in the basal region of the IHCs above the habenula (Figure 2F–J). The numbers in the epithelium remained elevated at 2 weeks recovery and also increased in base of the cochlea (Figure 2C,D,H). The macrophage numbers in the epithelium at 0 h recovery were insignificant from no exposure control (*p* > 0.05, two-way ANOVA) but the numbers in the epithelium at 2 weeks recovery were significantly different from no exposure control in the apex (*p* = 0.044), middle (*p* = 0.006) and base (*p* = 0.045) cochlear region (*F*_(2,55)_ = 21.93, two-way ANOVA). Macrophage numbers counted in the apex (5–11 kHz), middle (16–28 kHz) and base (32 and 45 kHz) IHC-ANF synaptic region (above the habenula) per 100 μm of the epithelium at 0 h were 2.8 ± 1.8, 4.2 ± 1.3 and 1.1 ± 0.75, respectively. Macrophage numbers in the IHC-ANF synaptic region at time 0 h recovery was significantly different from no exposure control only in the middle region (*p* = 0.0002, two-way ANOVA). By 2 weeks macrophage numbers in the apex, middle and base IHC synaptic region were 5.72 ± 0.6, 7.92 ± 1.6, and 3.42 ± 1.1, respectively. Those numbers were significantly different from no exposure control and 0 h recovery in all cochlear regions (*p* < 0.0001, *F*_(4,58)_ = 4.67, two-way ANOVA). These results indicate that moderate noise trauma that does not cause hair cell death is sufficient to induce a rapid and localized immune response into the vulnerable and damaged IHC synaptic region.

### CX_3_CR1^-/-^ Mice Exhibit Nearly Complete Recovery of Hearing Thresholds and OHC Function Following Moderate Noise Trauma

**FIGURE 3:**
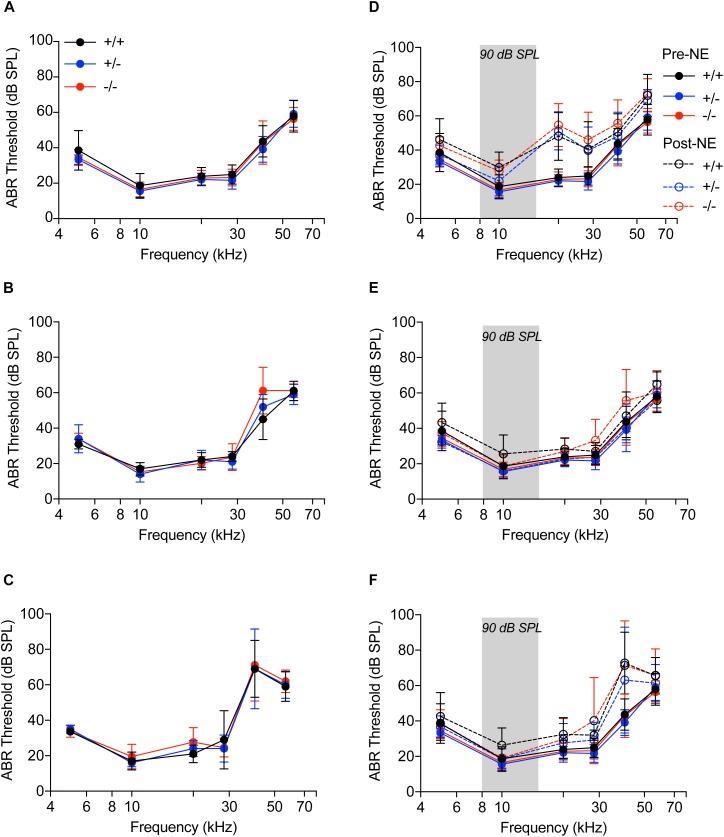
Auditory brainstem responses. **(A–C)** Audiograms of CX_3_CR1^+/+^ (black), CX_3_CR1^+/-^ (blue) and CX_3_CR1^-/-^ (red) mice not exposed to noise at 6 weeks **(A)**, 8 weeks **(B)**, and 14 weeks **(C)** of age. *n* = 5–6 mice per genotype. **(D–F)** Audiograms of CX_3_CR1^+/+^ (black), CX_3_CR1^+/-^ (blue), and CX_3_CR1^-/-^ (red) mice exposed to noise at time 0 h **(D)**, 2 weeks **(E)**, and 8 weeks **(F)** recovery. Filled circles with solid lines represent thresholds prior to noise-exposure (Pre-NE) and unfilled circles with hyphenated lines represents thresholds post-noise exposure (Post-NE). CX_3_CR1^+/+^ (*n* = 7), CX_3_CR1^+/-^ (*n* = 9), CX_3_CR1^-/-^ (*n* = 15). Gray bar in the graph represents the frequency band (8–16 kHz) of noise exposure.

We next sought to determine if cochlear macrophages influence auditory function, synaptic degeneration and repair after moderate noise trauma via fractalkine receptor, CX_3_CR1. Unexposed CX_3_CR1^+/+^, CX_3_CR1^+/-^, and CX_3_CR1^-/-^ mice had normal hearing thresholds at 6 weeks of age (Figure 3A). ABR thresholds were elevated at stimulus frequencies of 40 and 56.6 kHz in all genotypes at 8 weeks (Figure 3B) and at 14 weeks of age (Figure 3C) which is likely due to presbycusis in B6 mice. Therefore, any functional or structural data analysis at and above 40 kHz was excluded from the present study. ABR measurements immediately after exposure demonstrated a significant ∼20–30 dB elevation in hearing thresholds at stimulus frequencies above 20 kHz when compared to baseline thresholds in all the three genotypes (Figure 3D, *p* < 0.0001, *F*_(25,431)_ = 3.75, two-way ANOVA). The thresholds at 0 h were not different between the genotypes. The elevated thresholds returned to baseline by 2 weeks post-exposure (Figure 3E) and remained stable at 8 weeks (Figure 3F) except at higher frequencies. CX_3_CR1^-/-^ mice exhibit incomplete recovery of thresholds at 28.3 kHz region (Figure 3E,F) and were significantly elevated from baseline thresholds (*p* = 0.0056 at 2 weeks post-NE, *p* < 0.0001 at 8 weeks post-exposure, two-way ANOVA, Tukey’s *post hoc* multiple comparison test).

**FIGURE 4:**
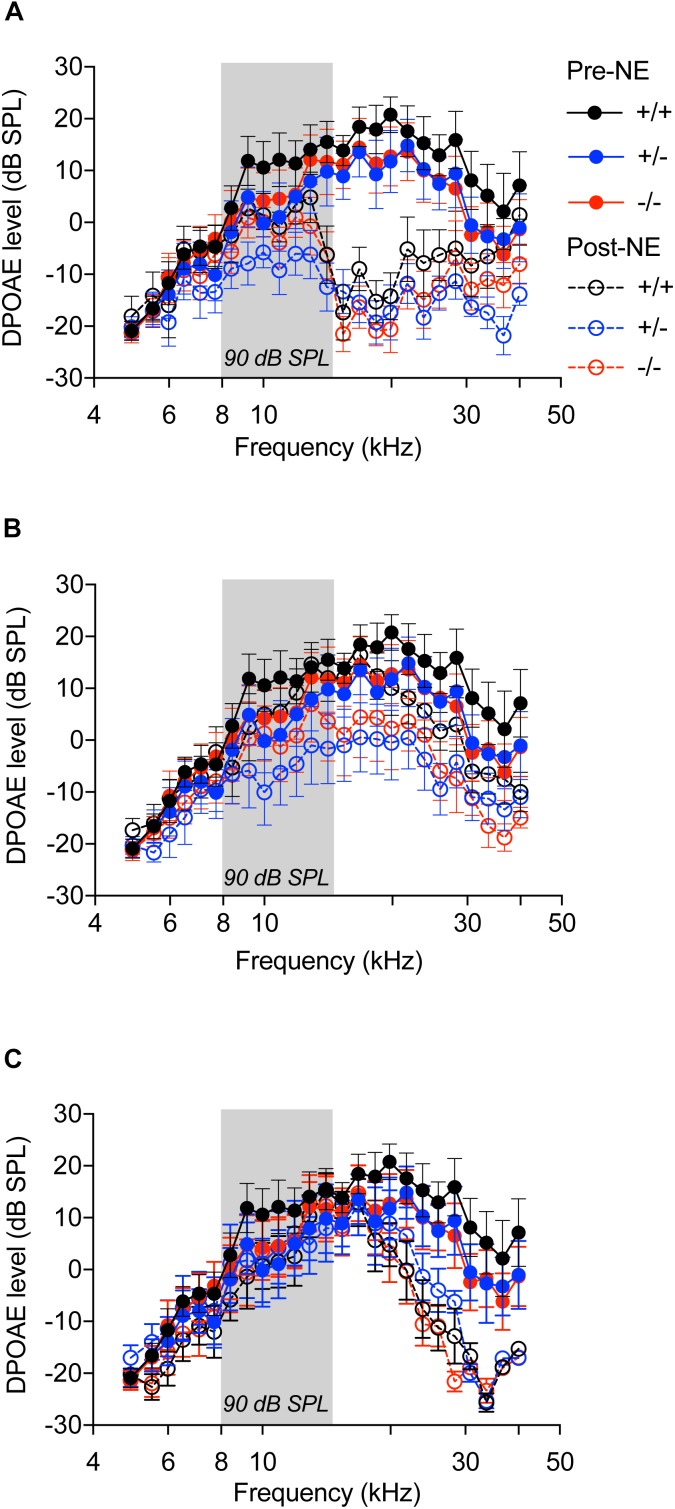
Distortion product otoacoustic emissions. DP grams of CX_3_CR1^+/+^ (black), CX_3_CR1^+/-^ (blue), and CX_3_CR1^-/-^ (red) mice exposed to noise at time 0 h **(A)**, 2 weeks **(B)**, and 8 weeks **(C)** recovery. Filled circles with solid lines represent DP grams prior to noise-exposure (Pre-NE) and unfilled circles with hyphenated lines represents DP grams post-noise exposure (Post-NE). CX_3_CR1^+/+^ (*n* = 8), CX_3_CR1^+/-^ (*n* = 9), CX_3_CR1^-/-^ (*n* = 9). Gray bar in the graph represents the frequency band (8–16 kHz) of noise exposure.

OHC function assessed by DPOAEs indicated no significant difference among the three genotypes prior to noise exposure (Figure 4A, filled circles with solid lines, *p* > 0.999, two-way ANOVA). Noise exposure immediately reduced DPOAE levels in all the genotypes (Figure 4A, unfilled circles with hyphenated lines, *p* < 0.0001, *F*_(120,1150)_ = 1.649, two-way ANOVA), when compared to baseline levels, with no significant difference among the genotypes. At 2 weeks after noise exposure, DPOAE levels returned to normal with no difference between the genotypes (Figure 4B). At 8 weeks, the DPOAE levels were reduced in all genotypes at frequencies above 20 kHz, however, the differences were not statistically significant (Figure 4C). These results demonstrate that lack of CX_3_CR1 does not influence hearing thresholds and OHC function after moderate noise trauma.

### CX_3_CR1^-/-^ Mice Display Attenuated ABR Wave 1 Amplitudes Following Noise Trauma

**FIGURE 5:**
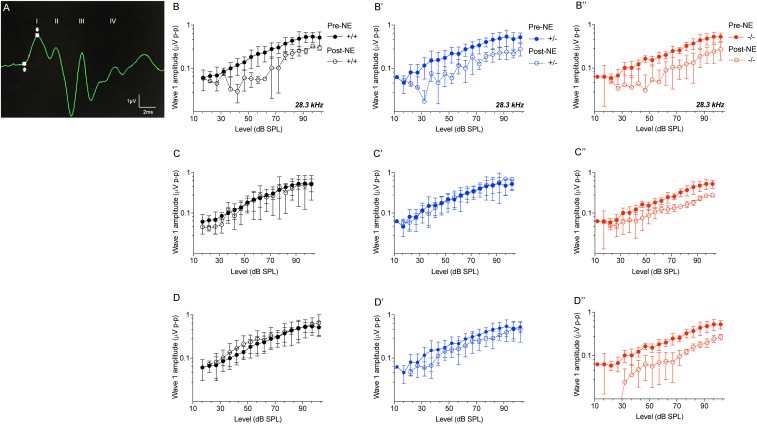
ABR Wave 1 amplitudes against level series. **(A)** Representative ABR trace (28.3kHz, 80 dB SPL) showing placement of arrows at peak and trough of ABR wave 1 for amplitude measurements. ABR wave 1 amplitudes at 28.3 kHz from CX_3_CR1^+/+^ (black), CX_3_CR1^+/-^ (blue), and CX_3_CR1^-/-^ (red) mice exposed to noise at time 0 h **(B–B″)**, 2 weeks **(C–C″)**, and 8 weeks **(D–D″)** recovery. Filled circles with solid lines represent amplitudes prior to noise-exposure (Pre-NE) and unfilled circles with hyphenated lines represents amplitudes post-noise exposure (Post-NE). CX_3_CR1^+/+^ (*n* = 7), CX_3_CR1^+/-^ (*n* = 9), CX_3_CR1^-/-^ (*n* = 15).

ABR wave 1 amplitudes-versus-stimulus level (ABR I/O) analysis at 28.3 kHz frequency region (Figure 5A) were significantly attenuated in all three genotypes immediately after noise exposure (0 h) compared to genotype matched pre-noise amplitudes (Figure 5B–B″, *p* < 0.01, two-way ANOVA). The attenuated wave 1 amplitudes recovered in CX_3_CR1^+/+^ and CX_3_CR1^+/-^ animals by 2 weeks after exposure and remained stable at 8 weeks (Figure 5C,C′,D,D′). However, CX_3_CR1^-/-^ mice displayed persistent attenuation in amplitudes (53%) at both thresholds as well as suprathresholds levels (Figure 5C″,D″, *p* = 0.015 and *p* = 0.0083 at 2 and 8 weeks post-exposure, respectively, two-way ANOVA). The raw amplitudes in CX_3_CR1^+/+^, CX_3_CR1^+/-^, and CX_3_CR1^-/-^ mice at 28.3 kHz are provided in Supplementary Table S1 (see Supplementary Material). ABR wave 1 amplitudes-versus-stimulus level (ABR I/O) analysis at 10 kHz frequency region revealed no difference in wave 1 amplitudes with respect to either genotype or recovery time post-noise exposure (data not shown). Together, the results show that exposed mice lacking CX_3_CR1 have attenuated amplitudes of ABR wave 1 when compared to exposed mice with intact fractalkine signaling.

### Lack of CX_3_CR1 Impairs Synaptic Repair and Influences IHC Survival After Noise Trauma

**FIGURE 6:**
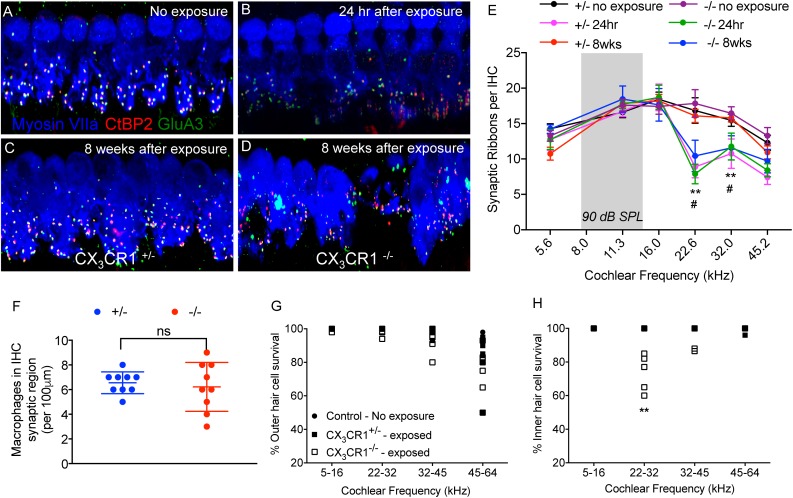
Ribbon synapses, macrophages, and hair cell numbers in CX_3_CR1^+/-^ and CX_3_CR1^-/-^ mice after noise exposure. **(A)** Representative micrograph from CX_3_CR1^+/-^ mice not exposed to noise (no exposure) at 32 kHz region immunolabeled for pre-synaptic marker CtBP2 (red), post-synaptic marker GluA3 (green) and inner hair cell marker Myosin VIIa (blue) showing intact juxtaposed ribbon synapses. **(B)** Representative micrograph at 24 h after exposure from CX_3_CR1^+/-^ mice at 32 kHz region showing disintegrated ribbon synapses. **(C)** Representative micrograph at 8 weeks after exposure from CX_3_CR1^+/-^ mice at 32 kHz region showing stable repaired synapses. **(D)** Representative micrograph at 8 weeks after exposure from CX_3_CR1^-/-^ mice at 32 kHz region showing degenerated ribbon synapses. Labeling colors in panels (**B–D**) are same as reported in A. **(E)** Ribbon synapses per surviving IHC along the cochlear frequency regions. No exposure; *n* = 6–7 mice per genotype, 24 h after exposure; *n* = 5–6 mice per genotype, 8 weeks after exposure; *n* = 9–15 mice per genotype. ^∗∗^*p* < 0.01 (CX_3_CR1^+/-^ and CX_3_CR1^-/-^, no exposure vs. 24 h), #*p* < 0.01 (CX_3_CR1^-/-^, 8 weeks vs. no exposure). Gray bar in the graphs represents the frequency band (8–16 kHz) of noise exposure. **(F)** Average macrophage numbers in IHC synaptic region per 100 μm of sensory epithelium of exposed CX_3_CR1^+/-^ and CX_3_CR1^-/-^ mice, *n* = 9 per genotype, ns, not significant. Percentage outer hair cell survival **(G)** and inner hair cell survival **(H)**. Mice per genotype not exposed to noise (*n* = 6), CX_3_CR1^+/-^ (*n* = 9), CX_3_CR1^-/-^ (*n* = 15) at 8 weeks after exposure. ^∗∗^*p* < 0.01. Scale, 17 μm (A-D).

To determine whether macrophages influence degeneration and repair of synapses via fractalkine signaling, CX_3_CR1^+/-^ and CX_3_CR1^-/-^ mice received a TTS-inducing noise exposure and temporal bones were collected at 24 h-, 2 weeks- and 8 weeks-recovery. Cochleae were fixed and processed for immunolabeling of presynaptic zones with an antibody against CtBP2, postsynaptic AMPA receptor subunit GluA3 antibody and for hair cells with anti-Myosin VIIa antibody (Figure 6A–D). There was no difference in ribbon synapses per surviving IHC across the cochlear length in unexposed (control) CX_3_CR1^+/-^ and CX_3_CR1^-/-^ mice (Figure 6E). At 24 h after exposure there was a rapid ∼50% loss of synaptic ribbons in both CX_3_CR1^+/-^ and CX_3_CR1^-/-^ mice throughout the basal turn of the cochlea (Figure 6B,E), compared to unexposed mice (Figure 6A,E). Two-way ANOVA followed by Tukey’s *post hoc* test revealed that synaptic counts were significantly reduced in exposed mice at the 22.6, and 32.0 kHz regions compared to unexposed control mice (*p* < 0.01, *F*_(25,208)_ = 1.79). There was no difference in synaptic counts between CX_3_CR1^+/-^ and CX_3_CR1^-/-^ mice at 24 h after exposure (Figure 6E, *p* = 0.99, two-way ANOVA). In exposed CX_3_CR1^+/-^ mice, damaged synapses recovered by 2 weeks post-exposure (data not shown) and remained stable at 8 weeks (Figure 6C,E). However, CX_3_CR1^-/-^ mice displayed persistent synaptic degeneration at the 22–32 kHz region at 2 weeks (data not shown) and 8 weeks after exposure (Figure 6D,E). Synaptic counts per surviving IHCs in CX_3_CR1^-/-^ mice at 8 weeks post-exposure were significantly different from unexposed CX_3_CR1^+/-^ and CX_3_CR1^-/-^ mice (*p* = 0.013, two-way ANOVA) but not significant from CX_3_CR1^+/-^ mice at 8 weeks recovery (*p* = 0.056). Lack of CX_3_CR1 did not affect macrophage numbers in the IHC-ANF synaptic region after exposure. The average macrophage numbers in the IHC synaptic region in exposed CX_3_CR1^+/-^ mice was 6.55 ± 0.88 and in exposed CX_3_CR1^-/-^ mice was 6.22 ± 1.98 (Figure 6F, *p* = 0.654, *t* = 0.46, Df = 11.4, two-tailed *t*-test). CX_3_CR1 deficiency also resulted in damage and loss of IHCs from the base of the cochlea of 40% mice (Figure 6H). IHC loss in exposed CX_3_CR1^-/-^ mice was significantly different from exposed CX_3_CR1^+/-^ and unexposed control mice (*p* = 0.0081, *F*_(6,108)_ = 3.08, two-way ANOVA). Noise-exposed mice had OHC loss in the base and hook regions in all three genotypes (Figure 6G), however the loss was statistically insignificant when compared to unexposed mice (*p* = 0.144, *F*_(6,108)_ = 1.67, two-way ANOVA). These results demonstrate that disruption of fractalkine signaling impairs the spontaneous recovery of damaged synapses after moderate noise trauma.

### CX_3_CR1 Deficiency Leads to Diminished Survival of SGNs Following Moderate Noise Trauma

**FIGURE 7:**
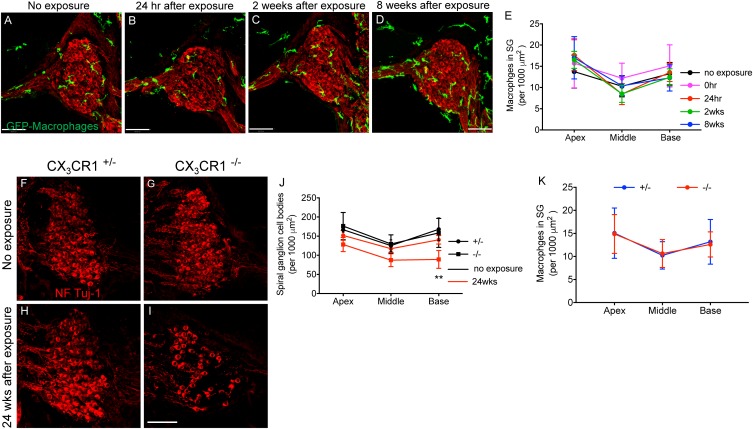
Macrophage and Spiral ganglion neuron density in the spiral ganglion of CX_3_CR1^+/-^ and CX_3_CR1^-/-^ mice after exposure. Representative micrographs of cochlear mid-modiolar sections from CX_3_CR1^+/-^ mice not exposed to noise (no exposure) **(A)** and from CX_3_CR1^+/-^ mice at 24 h **(B)**, 2 weeks **(C)**, and 8 weeks **(D)** after exposure immunolabeled for macrophages (green, GFP) and spiral ganglion neurons (red, NF165 and Tuj-1). **(E)** Macrophage numbers in spiral ganglion (SG) (per 1,000 μm^2^), *n* = 4–8 CX_3_CR1^+/-^ mice per recovery time point. Representative micrographs showing fewer SGN cell bodies immunolabeled for NF165 and Tuj-1 (red) in CX_3_CR1^-/-^ mice **(I)** compared to CX_3_CR1^+/-^ mice **(H)** at 24 weeks post-exposure and to unexposed age-matched control mice **(F,G)**. **(J)** Quantitative data on SGN density per 1,000 μm^2^ from age-matched unexposed and exposed CX_3_CR1^+/-^ and CX_3_CR1^-/-^ mice at 24 weeks post-noise trauma. *n* = 4 mice per group, ^∗∗^*p* < 0.01 CX_3_CR1^-/-^ mice 24 weeks post-NE vs. no exposure CX_3_CR1^+/-^ and CX_3_CR1^-/-^ mice. **(K)** Average macrophage density in SG of CX_3_CR1^+/-^ and CX_3_CR1^-/-^ mice at time 24 h, 2, 8, 16, and 24 weeks recovery after exposure. *n* = 4–9 per genotype per recovery time point. Scale bar, 63 μm.

Immunolabeling for macrophages using anti-GFP antibody (to enhance the fluorescence signal) in mid-modiolar frozen sections demonstrate that such moderate noise exposure for 2 h to an octave band (8–16 kHz) noise at 90 dB SPL did not result in an increase in macrophage numbers in the spiral ganglion (SG) at any recovery time point (Figure 7A–E). Two-way ANOVA followed by Tukey’s *post hoc* test revealed that macrophage density in the SG after noise exposure at any recovery time point was not significantly different from control or other recovery time points (*p* = 0.46, *F*_(8,78)_ = 0.977). To determine whether fractalkine signaling influenced SGN survival after synaptopathic TTS-like noise trauma, cochlear mid-modiolar frozen sections from CX_3_CR1^+/-^ and CX_3_CR1^-/-^ mice were immunolabeled for SGNs using NF165 and Tuj-1 antibodies. SGN density in exposed CX_3_CR1^-/-^ mice was indistinguishable to unexposed CX_3_CR1^-/-^ and exposed CX_3_CR1^+/-^ mice at 8 and 16 weeks post-exposure (data not shown). At 24 weeks after exposure, CX_3_CR1^-/-^ mice displayed increased loss of SGN cell bodies from the basal turn of the cochlea (Figure 7I,J) compared to unexposed CX_3_CR1^+/-^ and CX_3_CR1^-/-^ (Figure 7F,G,J) and exposed CX_3_CR1^+/-^ mice (Figure 7H,J). Two-way ANOVA followed by Tukey’s *post hoc* test revealed that the SGN density in the basal cochlear region of CX_3_CR1^-/-^ mice was significantly different from unexposed age-matched control mice (*p* = 0.0034, *F*_(10,57)_ = 6.22). The SGN density in the exposed CX_3_CR1^+/-^ mice was not statistically significant from the unexposed age-matched controls (*p* = 0.98). The macrophage density in the SG of CX_3_CR1^-/-^ mice was not different from that of CX_3_CR1^+/-^ mice at all recovery time point including 24 weeks after noise trauma (Figure 7K, *p* = 0.93, two-way ANOVA). These results demonstrate that disruption of fractalkine signaling due to genetic loss of CX_3_CR1 results in an increase in neuronal loss after moderate synaptopathic-noise trauma.

## Discussion

Cochlear synaptopathy can occur due to noise trauma (Kujawa and Liberman, 2009), ageing (Kujawa and Liberman, 2015), and aminoglycoside-induced ototoxicity (Ruan et al., 2014). Synaptic loss can precede threshold elevation and can trigger gradual degeneration of SGNs (Kujawa and Liberman, 2009). The mechanisms underlying synaptic damage and subsequent neurodegeneration have not been elucidated beyond the studies of glutamate excitotoxicity (Pujol et al., 1985, 1993). It is notable, however, that the ANFs can undergo spontaneous regeneration and can partially recover their synaptic connections with IHCs after excitotoxic insults (Puel et al., 1995; Pujol and Puel, 1999) or following acoustic trauma (Puel et al., 1998). Whether this represents transient protein down- and up-regulation or actual degeneration and regeneration of synaptic elements remains unclear. The mechanisms of such spontaneous synaptic repair are also unclear. In the present study young B6 mice exposed to 90 dB SPL for 2 h produced a TTS. The elevated thresholds, incomplete recovery of DPOAE levels and some degree of loss of OHCs observed at higher cochlear frequencies at 2 months post-exposure recovery is attributed to the early onset of hearing loss in B6 mice (Henry and Chole, 1980; Hequembourg and Liberman, 2001). We report that young B6 mice exposed to 90 dB SPL produces a rapid loss of up to 50% of synapses at cochlear regions tuned to frequencies higher than the exposure band (8–16 kHz). At 2 weeks post-exposure recovery, the damaged synapses undergo nearly complete spontaneous repair. This is in contrast to previous studies that reported permanent loss of synapses following moderate noise trauma that causes reversible threshold elevation (Kujawa and Liberman, 2009). Those studies utilized mice of CBA/CaJ strain at 16 weeks of age. Previous studies in guinea pigs demonstrated post-exposure regeneration of cochlear nerve terminals in the IHC region at the ultrastructural level (Puel et al., 1998). On the contrary, Lin et al., 2011 reported the presence of the irreversible primary neural degeneration in noise-exposed guinea pigs by immunolabeling pre- and post-synaptic markers and applying high power confocal analysis. The spontaneous synaptic recovery that has been reported in the present study may be attributed to the different strain, and age of mice as well as to the noise levels. We have found that young B6 mice exposed to 100 dB SPL causes permanent threshold shifts (PTS) and partial synapse recovery (data not shown). Similar post-exposure synapse recovery in B6 mice have also been demonstrated by Shi et al. (2015) and Kim et al. (2019). Further understanding of the mechanisms of spontaneous synaptic repair in B6 mice would lead to the identification of novel targets to elicit synapse regeneration.

Moderate noise trauma induced a rapid and focal recruitment of macrophages into the damaged synaptic region despite any evident hair cell death. The source and phenotype of macrophages as well as the mechanisms that regulate their recruitment into the synaptic region remain unknown. Studies from the CNS have reported that excitotoxity and/or neuronal injury can lead to microglial activation and chemotaxis toward the site of injury, and various candidate signals that could participate in microglial activation have been identified. Cytokines such as TNF-α, IL-1β, IFN-γ released from damaged neurons and from reactive astrocytes following excitotoxic events can activate the surrounding microglia (Zhang and Zhu, 2011). Lipid peroxidation (an oxidative stress indicator) plays an important role in neuronal degeneration. 4-hydroxynonenal, a product of lipid peroxidation, can activate and increase the phagocytic activity of microglia and peripheral macrophages through their association with scavenger receptors (Bruce-Keller, 1999). Molecules such as fractalkine have received much attention as microglial chemoattractants (Haskell et al., 1999; Hermand et al., 2008). CNS excitotoxicity can cause rapid cleavage of fractalkine from the neurons, increasing the levels of soluble fractalkine, which is a known chemoattractant (Chapman et al., 2000; Limatola et al., 2005). Nevertheless, our data show that the lack of CX_3_CR1 did not influence macrophage density in the IHC-synaptic region or in the spiral ganglion following moderate noise trauma, suggesting that fractalkine may not be a necessary chemotactic factor. Other molecules such as adenosine triphosphate (ATP), are released from injured cells, and evoke microglial chemotaxis to the injured site, acting via microglia P2Y12 purinergic receptors (Honda et al., 2001; Haynes et al., 2006; Eyo et al., 2014; Kato et al., 2016). Noise is known to stimulate local ATP release in the cochlea (Telang et al., 2010), however, whether ATP evokes macrophage recruitment into the synaptic region is unknown. Macrophages expresses glutamate receptors (Biber et al., 1999), hence it is possible that the excessive glutamate released from the IHCs due to noise trauma can directly induce macrophage chemotaxis toward the synaptic region. Glutamate can directly induce microglial chemotaxis which is mediated by AMPA and metabotropic glutamate receptors on the microglia (Liu et al., 2009). Future studies investigating the source and phenotype of macrophages and mechanisms that regulate their migration into the damaged synaptic region after noise trauma would be valuable to better understand the inflammatory cells dynamics in cochlear pathology.

Our data demonstrate that the genetic disruption of fractalkine signaling impairs the spontaneous recovery of damaged synapses and leads to enhanced degeneration of auditory neurons following synaptopathic noise trauma. The enhanced neurodegeneration in the damaged cochlea of CX_3_CR1 knockout animals corroborates our previous work (Kaur et al., 2015, 2018). The lack of synaptic recovery in the exposed CX_3_CR1^-/-^ mice is the first evidence for the role of macrophages and fractalkine in synaptic plasticity in the damaged cochlea. Increased synaptic degeneration in CX_3_CR1^-/-^ mice correlates with attenuated suprathreshold neural responses at higher frequencies without affecting hearing thresholds and DPOAE levels. Lack of CX_3_CR1 did not affect macrophage density in the IHC-ANF synaptic region or in the ganglion after exposure suggesting that the increased synapse degeneration and neuronal loss in exposed CX_3_CR1^-/-^ mice is not due to reduced macrophage numbers. The underlying mechanisms of synaptopathy in the CX_3_CR1 knockout animals is unclear, but could be driven, at least in part, by IHC pathology that is observed in a few (40%) CX_3_CR1^-/-^ mice. The exact contribution of macrophages in synaptic recovery is also not yet clear. An intriguing possibility is that macrophage migration into the damaged synaptic region is a protective mechanism designed to limit neurodegeneration and improve synaptic recovery following noise trauma. In support of this idea, serval studies in the CNS have suggested that microglial activation can attenuate excitotoxic injury and limit neurodegeneration (Simard and Rivest, 2007; Lauro et al., 2010; Vinet et al., 2012; Eyo et al., 2014; Kato et al., 2016) and improve synaptic recovery (Lazarov-Spiegler et al., 1996; Prewitt et al., 1997; Batchelor et al., 1999). Microglia-mediated protection against CNS excitotoxicity has been attributed to multiple mechanisms such as promoting neurite outgrowth (Prewitt et al., 1997; Batchelor et al., 1999), phagocytosing degenerating neurons and synapses (Abiega et al., 2016), and by promoting the release of neurotrophic factors such as basic fibroblast growth factor (bFGF) and nerve growth factor (NGF) (Heumann et al., 1987; Araujo and Cotman, 1993).

How might fractalkine signaling influence excitotoxicity and repair of cochlear synapses? One hypothesis is that fractalkine signaling regulates macrophage inflammatory and toxic behavior in damaged cochlea. Studies in the CNS have shown that fractalkine signaling exert neuroprotective actions in many neuroinflammatory and neurodegenerative disease models and can also prevent neuronal damage during glutamate excitotoxity (Cardona et al., 2006; Limatola and Ransohoff, 2014). The protective effect of fractalkine against glutamate excitotoxicity appears to involve a reduction in NMDA- or glutamate-mediated rise in intracellular calcium levels (Deiva et al., 2004; Sheridan et al., 2014), reduced AMPA type mediated currents (Meucci et al., 1998; Limatola et al., 2005; Ragozzino et al., 2006), increased glutamate removal from synaptic cleft by enhancing the expression of function of glutamate transporter-1 (GLT-1) on astrocytes (Catalano et al., 2013), and increased microglial adenosine release (Lauro et al., 2008; Cipriani et al., 2011). In addition, CX_3_CL1 can also modulate the clearance and phagocytic activity of microglia (Noda et al., 2011) that could be involved in the neuroprotective effects of CX_3_CL1 in CNS disorders. Mice lacking CX_3_CR1 develop increased disease severity in animal models of experimental autoimmune encephalomyelitis (EAE), low-endotoxemia, Parkinson’s disease, Amyotrophic lateral sclerosis, and Diabetic retinopathy (Cardona et al., 2006, 2015; Mendiola et al., 2017). To a certain degree, such pathology is attributed to enhanced microglial expression of inflammatory cytokines like IL-1β, TNF-α, and IL-6. Our previous work reported an increased expression of IL-1β in the injured cochlea of mice lacking CX_3_CR1 (Kaur et al., 2018). Future efforts toward understanding the mechanisms of fractalkine-mediated synaptic repair and neuroprotection may lead to therapeutic strategies for regeneration of synapses and neuroprotection in the injured cochlea.

Several animal studies have shown that neurotrophin therapies can partially regenerate damaged synapses and restore hearing after noise trauma (Wan et al., 2014; Suzuki et al., 2016; Chen et al., 2018). However, such partial effectiveness clearly demonstrates an additional need to understand the cellular and molecular mechanisms of neurite outgrowth and synapse regeneration to develop therapies to fully restore hearing. Our study has identified a novel endogenous immune pathway that may promote synaptic repair and neuron survival during hearing loss. Future studies investigating the contribution of activation of fractalkine signaling in degeneration and repair of cochlear synapses and determining the function of each fractalkine isoform (membrane or soluble) are necessary to completely understand the role of fractalkine in cochlear synaptopathy and neuropathy. Our research work also poses important clinical relevance for humans carrying the polymorphic variant CX_3_CR1^I249/M280^ that is estimated to be present in about 20–30% of the population. These changes in human CX_3_CR1 decrease CX_3_CL1 affinity (McDermott et al., 2001) and have been associated with multiple neurodegenerative disorders such as age-related macular degeneration (Chan et al., 2005; Schaumberg et al., 2014), Alzheimer’s disease (Lopez-Lopez et al., 2017), and multiple sclerosis (Arli et al., 2013; Cardona et al., 2018). Based on the evidence of enhanced synaptic and neuronal damage in the injured cochlea of CX_3_CR1-deficient mice, it would be of clinical relevance to dissect the effects of human CX_3_CR1^V249/T280^ receptor and its polymorphic variant CX_3_CR1^I249/M280^ in deaf ears. Such studies may merit evaluation as risk factors for susceptibility to sensorineural hearing loss. Together, our data suggest a pivotal role of CX_3_CL1-CX_3_CR1 signaling in repair of damaged synapses and neuron survival after noise trauma. Thus, modulating fractalkine signaling may be a relevant approach to mitigate cochlear synaptopathy and neuropathy.

## Data Availability

The datasets generated for this study are available on request to the corresponding author.

## Ethics Statement

This study was carried out in accordance with the recommendations of the Animal Welfare Act and Animal Welfare Regulations issued by the United States Department of Agriculture (USDA). All experimental protocols were approved by the Institutional Animal Care and Use Committee of the Washington University School of Medicine (St. Louis, MO, United States).

## Author Contributions

TK and KO designed and planned the study. TK, AC, AN, and KO performed the experiments and analyzed the data. AS performed the mouse genotyping. TK, MW, and KO contributed to the writing and editing of the manuscript. All authors read and approved the submitted version of the manuscript.

## Conflict of Interest Statement

The authors declare that the research was conducted in the absence of any commercial or financial relationships that could be construed as a potential conflict of interest.

## Supplementary Material

TABLE S1 Raw values of ABR wave 1 amplitudes against level series.
